# PP15 Cost–Benefit Analysis Of An Antimicrobial Stewardship Program In A Cancer Setting In Qatar

**DOI:** 10.1017/S0266462324001880

**Published:** 2025-01-07

**Authors:** Dina Abushanab, Diala Alhaj Moustafa, Bhagyasree Adampally Sankar, Ziad Nasr, Moza Al Hail, Anas Hamad, Palli Valappila Abdul Rouf, Wessam Elkassem, Binny Thomas, Daoud Al-Badriyeh

## Abstract

**Introduction:**

The inappropriate use of antimicrobial agents has been associated with increased healthcare costs and the spread of multidrug-resistant organisms. We aimed to estimate the economic impact of the developed antimicrobial stewardship program (ASP), after five years of implementation, versus the preliminary ASP, upon implementation, in the cancer setting at the National Center for Cancer Care and Research (NCCCR) in Qatar.

**Methods:**

The research investigated the economic benefits of employing a preliminary ASP versus a developed ASP from the perspective of a public healthcare hospital. Preliminary ASP was defined as the 12 months following the establishment of the ASP (i.e., 1 May 2015 to 30 April 2016), while developed ASP was defined as the most recent 12 months of ASP implementation (i.e., 1 February 2019 to 31 January 2020). Patient records were retrospectively reviewed. The total economic benefit of ASP maturity was calculated as the sum of the cost savings and the cost avoidance associated with the service, minus the operational cost.

**Results:**

A total of 1,000 patients were included in the study. The developed ASP was associated with substantial reduction in antimicrobial consumption and resource utilization. Total cost of resources to avoid during the developed ASP period was USD1,634,658, in contrast to USD4,923,024 during the preliminary ASP period, yielding a positive cost avoidance of USD3,288,366. Developed ASP incurred lower operating costs than preliminary ASP, resulting in positive change in operational cost of USD3,428. The benefit-to-cost ratio was 640. The net benefit due to ASP maturity was USD3,624,875. The robustness of the results is demonstrated by the sensitivity analysis.

**Conclusions:**

This study underscores the benefits of ASP development in a cancer setting. The observed reductions in antimicrobial costs from reduced antimicrobial use and the added value of cost avoidance signify the positive impact of a well-developed ASP. These findings lend support to the broader implementation of comprehensive ASPs, which contribute not only to patient-care optimization but also to cost containment.

